# The New York Physicians’ Mutual Aid Association

**Published:** 1886-08

**Authors:** 


### *THE NEW YORK PHYSICIANS' MUTUAL AID ASSOCIATION.*

This association, whose work has heretofore been limited to the profession of New York and Brooklyn, has decided to extend its privileges and benefits to the profession of the entire State. We desire to call attention to it, as we believe the association merits the support of every physician. Any member of the regular profession, under fifty years of age, in good health and in actual practice, or a teacher or professor in a medical school, is eligible to membership. The cost of becoming a member is three dollars—two dollars for the initiation fee and one dollar for the first assessment. Assessments, one dollar each, are made upon the death of a member; if a member has been admitted when over fifty years of age, his assessment is two dollars.

Equally important with its life-insurance feature, is the benevolent part of its work. The interest of a permanent fund is used for the relief of cases of sickness or destitution occurring among its members or their heirs. This permanent fund has now reached nearly $14,000, the latest contribution to it being that of Mrs. Austin Flint, of $450.

The sevententh annual report of the association has just been published, which can be obtained from the secretary, Dr. W. Y. Alexander, Station M, New York City, or of any of the following gentlemen, who have been appointed examiners for the State-at-large: Dr. H. Flood, Elmira; Dr. S. Ely, Newburg; Dr. W. W. Hewlett, Babylon; Dr. C. M. Wilson, Gouverneur; Dr. W. E. Ford, Utica; Dr. W. S. Ely, Rochester; Dr. F. H. Potter, Buffalo.

---

*To the Editor of the Buffalo Medical and Surgical Journal:*

DEAR SIR—In the last issue of your JOURNAL, in which appears a report of the proceedings of the last meeting of the Central New York Medical Association, you have given a brief abstract of my address (as retiring president,) on "Differentiation in Medicine, the Necessary Results of the Law of Evolution."

In this abstract, on page 569, you have made me quote Dr. George Johnson, of London, as follows: "Of the specialist, it should be said with truth that he is not one who knows more of the particular class of diseases to which he devotes most time and especial attention and study."

The omission from the above of a portion of the quotation has quite altered the sense of what Dr. Johnson says. The correct quotation is as follows: "Of the specialist, it should be said with truth that he is one, not who knows less of the disease in general, but who knows more of the particular class of disease to which he has devoted most time and special attention and study." (*Vide* transactions of the International Medical Congress, London, 1881, vol. iii, p. 196. Inaugural address, by the chairman, before the section of the diseases of the throat.) I am, sir,

Yours faithfully,

JOHN O. ROE, M. D.

Rochester, July 15, 1886.

